# Brain–body interactions associated with the transition from mind wandering to awareness of its occurrence

**DOI:** 10.1093/nc/niaf059

**Published:** 2025-12-15

**Authors:** Kazushi Shinagawa, Yuto Tanaka, Yuri Terasawa, Satoshi Umeda

**Affiliations:** Keio University Global Research Institute, 2-15-45 Mita, Minato-ku, Tokyo 108-0073, Japan; Department of Information Medicine, National Center of Neurology and Psychiatry, 4-1-1 Ogawa-higashi-cho, Kodaira-shi, Tokyo 187-8551, Japan; Japan Society for the Promotion of Science, Kojimachi Business Center Building, 5-3-1, Kojimachi, Chiyoda-ku, Tokyo 102-0083, Japan; Keio University Global Research Institute, 2-15-45 Mita, Minato-ku, Tokyo 108-0073, Japan; Department of Psychology, Keio University, 2-15-45 Mita, Minato-ku, Tokyo 108-0073, Japan; Keio University Global Research Institute, 2-15-45 Mita, Minato-ku, Tokyo 108-0073, Japan; Department of Psychology, Keio University, 2-15-45 Mita, Minato-ku, Tokyo 108-0073, Japan

**Keywords:** mind wandering, awareness, heartbeat-evoked potentials, respiration, self-caught methods, interoception

## Abstract

Our thoughts are inherently dynamic and often wander far from our current situation (mind wandering, MW). Although previous research revealed that brain regions involved in arousal regulation modulate neural dynamics to facilitate the transition from MW to the awareness of its occurrence, the specific physiological states and afferent signals underlying this process remain unclear. In this study, we examined electroencephalography, electrocardiography, and respiration data before participants were aware of MW during a task in which they focused on external or internal stimuli (tones or their breath). We showed that the transition to awareness of MW was characterized by decreased alpha and beta activity and a suppression of the parietal later component of the heartbeat-evoked potential (HEP), a modulation pattern identified in this study as a marker of enhanced central processing based on its consistent appearance across internal attention conditions. Furthermore, when participants were instructed to focus on their breath, they were more likely to be in the exhalation phase during the transition to awareness of MW and in the inhalation phase when MW was reported. This respiratory pattern was accompanied by changes in cardiac activity and HEP amplitudes. Based on these findings, we propose that the transition from MW to the awareness of its occurrence is associated with diverse neural activity, including the enhanced processing of bodily signals that co-occurs with specific cardiac and respiratory dynamics.

## Introduction

The human brain dynamically generates conscious experiences, which encompass various aspects of our subjective experiences. Humans spend a large proportion of these experiences engaged in mind wandering (MW; Marcusson-Clavertz *et al.* 2023; Spronken *et al.* 2016), which is often defined as self-generated thoughts that are decoupled from the current environment or task (Smallwood and Schooler 2015). To reduce the time spent engaging in MW in undesirable situations (e.g*.* at work or in class), it is important to become aware of MW immediately after it occurs (Sayette *et al.* 2009). The inability to monitor and disengage from excessive or distracting self-generated thoughts is associated with impairments in well-being (Andrews-Hanna *et al.* 2014). However, the underlying mechanisms leading to awareness of MW, a kind of change in conscious experience, are not yet fully understood.

In our daily lives, the brain generates cognitive experiences both on its own and through interactions with the body (Park and Tallon-Baudry 2014; Criscuolo *et al.* 2022). Recent research on the dynamics of conscious experience has emphasized brain–body interactions (Carriere *et al.* 2013; Northoff 2018; Candia-Rivera 2022; Signorelli *et al.* 2022), with empirical evidence highlighting the contributions of various physiological modalities. For instance, cardiac signals also contribute to conscious experiences, as demonstrated by associations between the rigidity of thoughts and cardiac activity (Ottaviani *et al.* 2013) and between thought self-relevance and cortical responses to it (Babo-Rebelo *et al.* 2016). Heartbeat-evoked responses in electroencephalography (EEG) signals are more accurate at distinguishing consciousness from unconsciousness than random EEG signals are (Candia-Rivera *et al.* 2021). Considering the evidence that cardiac activity modulates perception and emotion (Park and Tallon-Baudry 2014; Nguyen *et al.* 2016; Salomon *et al.* 2016), afferent signals stemming from cardiac activity may contribute to various aspects of conscious experiences. Additionally, respiration is closely related to the locus coeruleus (LC), which supports sustained attention (Allen *et al.* 2023). Respiration affects heart rate physiologically, and respiration phases modulate the central cardiac activity process (Zaccaro *et al.* 2022). Taken together, these findings support the potential effects of brain–body interactions on conscious experiences, including the awareness of the occurrence of MW.

Although there are no direct findings, previous research on MW has suggested the role of physiological activity during the transition from MW to awareness of its occurrence. In a recent study, changes in neural activity during conscious experiences were tracked *via* the self-caught method (Munn *et al.* 2021). In this method, participants reported whenever they noticed MW (Smallwood and Schooler 2006), allowing researchers to extract the shift from MW to awareness of its occurrence. The findings revealed that brain regions associated with arousal regulation become active several seconds before MW is reported (Munn *et al.* 2021), a process thought to modulate the excitability and receptivity of neurons across the brain (Wainstein *et al.* 2022). These results suggest that autonomic activity can regulate ongoing brain dynamics, supporting changes in conscious experiences (Candia-Rivera 2022). However, although autonomic activity may influence the transition from MW to awareness of its occurrence, the specific bodily state changes and related neural processing remain unclear.

**Figure 1:**
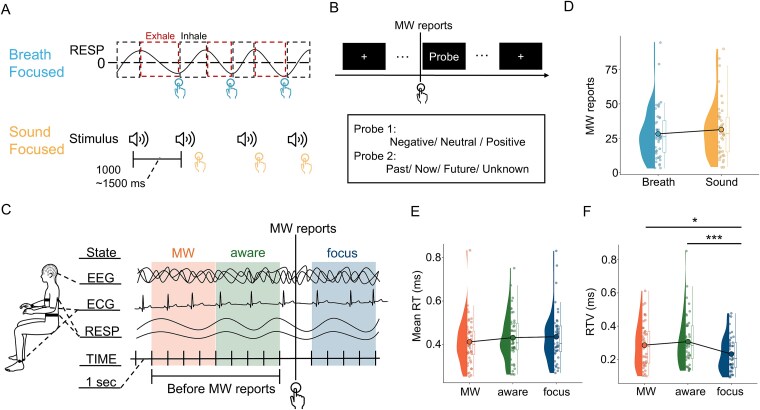
Experimental settings and behavioral results. (A) and (B) Overview of the experimental design: In the breathing-focused condition, the participants responded at the end of exhalation, and in the sound-focused condition, they responded to each sound. During the response task, participants reported when they became aware of MW and answered questions about the content of their thoughts (emotional valence and time orientation) before resuming the task. Sound stimuli were consistently presented in both conditions. Each task was performed for four 12-min sessions. (C) Measurement indices and definitions of attentional states: We recorded EEG (65 channels), ECG (1 channel), and respiration (2 channels) data during the task. The interval spanning from 1 to 5 s before the MW report was used to define the aware state, whereas the period spanning from 5 to 9 s before the MW was reported was defined as the MW state. The focus state was defined as the 4 s after the initial sound stimulus after the task was resumed. (D) MW frequency per task condition: The horizontal axis shows the task condition, and the vertical axis shows the average number of MW reports. Each dot represents the data for one participant. (E) and (F) Reaction time and its variability among attentional states: The horizontal axis shows the attentional state, and each dot represents the mean value for one participant. ^*^P < .05, ^**^P < .01, and ^***^P < .001.

In the present study, we aimed to investigate the dynamics of brain activity and the physiological state that leads to the transition to awareness of MW. We combined a simple reaction task with the self-caught method and recorded EEG, electrocardiogram (ECG), and respiration data (Fig. 1A–C). The reaction task included two conditions: participants responded either to continuously presented sounds (sound-focused; SF) or to their own exhalations (breathing-focused; BF). The data acquired before and after the MW reports were segmented into three states to investigate changes related to the transition to awareness of the MW (Fig. 1C). The MW state, in which participants were immersed in their internal thoughts, was defined as the period 5–9 s before MW was reported, on the basis of previous studies (Braboszcz and Delorme 2011; Bastian and Sackur 2013; Polychroni *et al.* 2022). The aware state was defined as the interval during which brain activity and physiological signals transitioned from MW to awareness of its occurrence, occurring 1–5 s prior to reports of MW. These definitions are grounded in findings that increased activity of the brain regions related to arousal and external perceptual processing—which may reflect a departure from MW—occurs 3–5 s before MW is reported (Munn *et al.* 2021; Shinagawa *et al.* 2021). To further characterize the MW state, we defined the focus state as the 4-s interval beginning with the presentation of the first sound stimulus following task resumption, during which participants concentrated on external stimuli (SF) and respiration (BF). In our study, participants were instructed to refocus on the task as soon as it resumed, similar to previous studies (Braboszcz and Delorme 2011; Bastian and Sackur 2013; Polychroni *et al.* 2022). Thus, the interval after task resumption was defined as the period during which participants maintained their concentration on the task.

We first conducted analyses to validate our experimental paradigm. Consistent with previous findings, we hypothesized that the MW state, compared with the focused state, would be characterized by (1) increased or variable reaction times, (2) modulations of oscillatory power (primarily an increase in lower frequency and a decrease in higher frequency), and (3) changes in autonomic indices. In addition, we hypothesized that heartbeat-evoked potentials (HEP) amplitudes would increase, especially when attention was directed toward breathing.

We subsequently tested our primary hypotheses regarding the transition to awareness of MW occurrence. The hypothesis was that the transition would be characterized by a decrease in the oscillatory power that was enhanced during the MW state. We predicted that the central cardiac signal process, as represented by HEP amplitudes, would also be enhanced during the aware state, similar to the pattern observed during the breath-focused state. Following the testing of these primary hypotheses, we also explored the temporal dynamics of these neural and physiological changes.

Furthermore, while the transition itself is our primary focus, we also exploratorily hypothesized that the MW state was influenced by brain–body interactions. A behavioral study indicated that heart rate manipulation caused participants to be more likely to continue engaging in self-referential thought during MW. This tendency interacted with how accurately the participants could feel their own heartbeat, suggesting that physiological changes and their brain’s ability to sense them influence the orientation of the contents during MW (Sakuragi *et al.* 2023). Additionally, it has been shown that heartbeat-evoked responses within the default mode network—a brain network associated with MW—are related to the self-relatedness of thoughts during MW (Babo-Rebelo *et al.* 2016). Therefore, we also aimed to characterize how brain–body physiology is related to the MW state and its content. The detailed methods and results for this hypothesis are provided in the Supplementary Materials.

## Materials and methods

### Participants

We recruited 44 healthy university students with normal or corrected-to-normal vision and hearing who were located in Tokyo (24 women; mean age = 21.21; *SD* = 1.96; *range* = 18–29). None of the participants had a history of mental or neurological disorders. This study was approved by the Keio University Research Ethics Committee (No. 18006) and was conducted in accordance with the Declaration of Helsinki. All participants provided written informed consent prior to participation. The participants were paid 7 000 JPY.

The sample size was determined by an *a priori* power analysis. The analysis was based on robustly reported behavioral correlates of MW, including reaction time variability (RTV). On the basis of previous research, we anticipated a medium effect size (Cohen’s d = 0.5) for the difference in RTV between MW and focused states (Seli *et al.* 2013). With a desired power of (1–β) = 0.80 and a significance level of α = 0.05 (two-tailed), the analysis indicated that a minimum of 34 participants were needed. To account for potential participant exclusions from physiological analyses (e.g*.* owing to an insufficient number of MW reports), we recruited a larger sample of 44 participants.

### Apparatus

In this study, visual stimuli were displayed on a PC monitor placed in front of the participants, whereas auditory stimuli were delivered through speakers located ~0.75 m from either side of the participants at a 45° angle. The participants' responses were collected *via* a keyboard.

EEG data were acquired at a sampling rate of 500 Hz using a 64-channel HydroCel Geodesic Sensor Net connected to a high-input impedance amplifier (Net Amps 300, Electrical Geodesics Inc., Eugene, OR, USA), and the signals were referenced to the vertex (Cz). The electrode impedance was adjusted as needed during intersession intervals and maintained below 50 kΩ. For ECG data collection, Ag/AgCl electrodes were attached to the back of the right hand and ankle of each participant. For respiratory data collection, we recorded chest wall and abdominal excursions *via* inductance plethysmography, which allowed us to determine the respiratory phase (inhalation or exhalation) during the task (Ambu, Ballerup, Denmark and Sleepmate or Perfect Fit 2, Dymedix, St Paul, MN, USA). EEG, ECG, and respiratory data acquisition was controlled by NetStation software (v. 5.3.0.1; EGI, Inc. Eugene, OR, USA). The presentation of the stimuli and the collection of participant responses were controlled *via* a stimulus delivery and experiment control program (Presentation; Neurobehavioral Systems, Inc., Berkeley, CA, USA).

### Experimental design and task

#### Procedure

In the present study, the participants completed a heartbeat counting task (HCT) and two simple response tasks. We used the HCT to measure participant-specific interoceptive accuracy (IAcc), which is an objective accuracy metric for detecting internal bodily sensations (Garfinkel *et al.* 2015). Analysis of the HCT results is not the main purpose of this study and is therefore included in the Supplementary Materials. Two simple response tasks conducted using the self-caught method were used to compare differences in attentional states. EEG, ECG, and respiratory data were measured during these response tasks. Because of the time required to complete the response tasks, they were conducted over two days to reduce participant fatigue. The HCT was always performed on the first day, and the response tasks were counterbalanced among the participants regarding the order in which they were performed.

#### Heartbeat counting task

A pulse oximeter (NONIN, 8600) was placed on the left index finger of each participant. In the HCT, we instructed the participants to silently count their heartbeats at different intervals without taking their pulse or changing their breathing. In addition, participants were reminded to respond with the number of times they actually felt a heartbeat rather than guessing. This task was repeated six times with time windows of 25, 30, 35, 40, 45, and 50 s, presented in randomized order. The participants began counting after hearing ‘start’ and stopped counting after hearing ‘stop’. After counting, the participants reported the number of heartbeats they counted and their confidence in their answers *via* a keyboard. This task was conducted using ‘cardioception,’ a Python package (Legrand *et al.* 2022). The absolute proportional difference between the number of reported and actual heartbeats was computed to quantify the cardiac IAcc (Eq. 1).


(1) \begin{equation*} 1-\frac{\left| Actual- Reported\right|}{Actual} \end{equation*}

#### Two simple response tasks with the self-caught method

The participants performed the two simple response tasks in a dimly lit, soundproof room, and EEG, ECG, and respiratory data were recorded. The task included a sound stimulus response condition (sound-focused; SF) and a breathing response condition (breathing-focused; BF).

In the SF condition, the participants were asked to press the key immediately after each continuously presented pure tone. We randomly changed the interstimulus interval (between 1.2 and 2 s) to prevent habitual responses. To reduce habituation to the auditory stimuli, a set of 50 unique pure tones with different frequencies (ranging from 400 to 1400 Hz in 20 Hz intervals) was created. For each stimulus presentation, a tone was randomly selected from this set. Each sound was presented for 100 ms. The tone was presented at 65 dB. In the BF condition, the participants were asked to press the key after each end of exhalation. Pure tones were also presented in the BF condition to remain consistent with the environment in the SF condition.

The participants were instructed to report instances of MW whenever they were aware of their thoughts drifting away from the task at hand, guided by the explanations provided before task execution (Smallwood and Schooler 2006). In the present study, MW was defined as the occurrence of thoughts unrelated to the task and stimulus (Stawarczyk *et al.* 2011; Smallwood and Schooler 2015). To facilitate their understanding and recognition of MW, we presented examples of both task- and stimulus-related thoughts, such as ‘How many tones were presented?’ and ‘This tone is C,’ as well as thoughts unrelated to the task and stimulus, such as ‘What shall I have for lunch today?’ and ‘Something upsetting happened yesterday.’

The participants responded to the sound stimuli or the end of exhalation by pressing the ‘1’ key on the keyboard and reported MW by pressing the ‘3’ key. After MW was reported, we asked two questions to capture the content of the participants’ thoughts. First, regarding the emotional valence of the thoughts, the participants used the keyboard to indicate whether their thoughts were negative (“4”), neutral (“5”), or positive (“6”). Second, concerning the temporal aspects of their thoughts, the participants indicated whether these thoughts were related to the past (“4”), present (“5”), or future (“6”). If the thoughts lacked a temporal aspect or if the participants were uncertain, they were instructed to press “8”. After responding, the participants resumed the task at their own pace by pressing the enter key. Analysis of thought content during MW is not the main purpose of this study and is therefore included in the Supplementary Materials.

Each task condition lasted ~1 h and included periods of rest between the four 12-min sessions. In addition, a ‘+’ symbol was displayed in the center of the screen, and the participants were asked to look at the symbol during the task. A schematic representation of the task workflow is presented in Fig. 1A.

#### Preprocessing and statistical analysis

To clarify the mechanism underlying spontaneous awareness of MW occurrence, we focused mainly on neural activity, the physiological state, and the central cardiac activity process during the above thought states. Time–frequency analysis was performed on the basis of the EEG data to examine overall neural activity during the transition to awareness of MW. In addition, we examined physiological states from cardiac activity and respiratory phases, which may influence the conscious experience of thought. As indices of these central processes, we utilized HEP, an EEG component time-locked to the R-peak of the heartbeat (Schandry *et al.* 1986). The HEP are considered to reflect afferent signals from the heartbeat and processing in the brain, and many studies have utilized HEP to investigate how the cardiac activity process influences cognitive functions (Coll *et al.* 2021). A recent study revealed that heartbeat-induced pulsations of cerebral blood vessels can directly affect central neuronal activity by activating mechanosensitive channels, supporting the physiological validation of HEP (Jammal Salameh *et al.* 2024). By comparing these indices in attentional states, we investigate how brain–body dynamics lead to awareness of MW.

#### Definitions of attentional states

In our study, we categorized participants' experiences into three distinct states: MW (MW state), transitioning to awareness of MW (aware state), and focused attention on the main task (focus state). Previous studies using self-report methods have suggested that participants’ minds wandered for ~9 s before their MW was reported (Braboszcz and Delorme 2011; Bastian and Sackur 2013; Polychroni *et al.* 2022). Furthermore, other studies revealed that the transition from MW to awareness begins 3–5 (or 4) s before MW is reported (Munn *et al.* 2021; Shinagawa *et al.* 2021). Based on these findings, we defined the period 5–9 s before the self-report as the MW state and 1–5 s before the self-report as the aware state. The second immediately preceding the report was excluded to minimize the influence of myoelectric potentials associated with MW reporting. While the subjective experience of becoming aware of MW is likely an instantaneous event, we defined the aware state using a time window. We designed this time window specifically to capture the gradual neurophysiological changes that precede the discrete moment of awareness. This approach of defining an `AWARE phase' preceding the report of MW is consistent with prior work (Hasenkamp *et al.* 2012).

The focus state was defined as the 4-s interval beginning with the presentation of the first sound stimulus after task resumption, as illustrated in Fig. 1B. During this interval, participants were required to concentrate on either external auditory stimuli (SF) or their breathing (BF). Participants were explicitly instructed to promptly refocus on the task as soon as it resumed, similar to previous studies (Braboszcz and Delorme 2011; Bastian and Sackur 2013; Polychroni *et al.* 2022). Thus, the period after task resumption was operationalized as the time during which participants maintained their focused attention on the task.

To ensure the spontaneity of the reports of MW, we placed no limits on the number or timing of MW reports. However, if participants reported multiple instances of MW within a short period, the time window was labeled both the MW and task state. We excluded MW reports that occurred within 10 s of the preceding MW report in the EEG analysis. This approach was taken to ensure that the attentional state was accurately identified.

#### Respiratory data

All the physiological data were preprocessed using MATLAB R2023b (MathWorks Inc. Natick, MA, USA). Respiratory data were used to evaluate associations between awareness of MW and the respiratory phase. Raw data preprocessing and respiratory phase detection were performed *via* the BreathMetrics toolbox (Noto *et al.* 2018). Before the raw breathing flow recordings were decomposed into phase data, measurement noise and signal drift were removed. First, the signal was mean-smoothed by a 25-ms window. Next, global linear drift was removed by subtracting the slope of the linear regression model of the data. Local signal drifts were corrected to continuous, minute-long sliding mean baseline windows, and padding was removed. These processes and parameters are default settings in the package. The respiratory phase was then detected at each time point on the basis of the processed respiratory data. In this study, respiratory data from the chest and abdomen were acquired to collect thoracic and abdominal breathing data from the participants. Since thoracic breathing involves less abdominal movement, we selected the respiratory data that showed more cycles for each participant for subsequent analyses.

Respiratory data were used to evaluate associations between the MW reports and the respiratory phase. While circular statistics are a standard approach for analyzing cyclical rhythms, our analysis was designed to test the specific hypothesis that the physiologically distinct states of inhalation and exhalation, associated with sympathetic and parasympathetic activity, respectively, would show different patterns of associated cardiac activity (RR intervals) and central processes (HEP) preceding the awareness of MW. To test this hypothesis, it was necessary to first categorize the respiratory cycle into discrete 'inhalation' and 'exhalation' phases to label each cardiac event. This approach of comparing physiological measures between binned respiratory phases, although different from circular statistics that test for phase concentration, is an established method used in similar prior work (Zaccaro *et al.* 2022). To achieve this comparison, we first established a baseline inhalation rate for each participant by creating an empirical null distribution from 10 000 random samples from their entire respiratory phase sequence. We then employed a series of one-sample t tests to assess whether the observed inhalation rate at each second before an MW report significantly deviated from this baseline. The percentages of inhalation were 0.651 and 0.615 in the BF and SF conditions, respectively, and we tested whether the inhalation rate every second before MW was reported was more or less than the baseline value in each condition. The inhalation rate was calculated by dividing the number of inhalations at a given time point by the total number of MW reports. Additionally, we used a paired t test to compare the number of respiratory cycles between task conditions to help elucidate how the task type affects breathing patterns.

#### Electrocardiogram

ECG data were used for HEP calculations and to investigate physiological changes depending on the attentional state. For this purpose, we first preprocessed the raw ECG signals by applying a 3–30 Hz bandpass filter. We then extracted R peaks using the heplab_slowdetect function included in HEPLAB (Perakakis 2019), an EEGLAB extension for automatically detecting cardiac-related events from ECG signals. Since this function cannot identify all the peaks and may incorrectly identify noise showing abrupt changes, such as R peaks, we corrected instances of misidentification *via* visual inspection. The R peak timing data were used as events for the HEP calculation.

Following R peak detection, beat-to-beat RR intervals were calculated. To compare cardiac activity across attentional states, each RR interval was assigned to the state (MW, aware, or focus) in which it occurred. To obtain a stable estimate for each attentional state by minimizing noise from individual segments, we averaged all RR intervals across all corresponding epochs for each participant. This ensemble averaging approach is supported by prior work on short-term HRV analysis (Munoz *et al.* 2015).

### Electroencephalography

#### Preprocessing

All EEG data preprocessing, HEP calculations, and time–frequency analyses were performed *via* EEGLAB v2023.1 for MATLAB (Delorme and Makeig 2004). First, all the acquired raw data were preprocessed for HEP and time–frequency analysis. The recording frequency was 500 Hz, but since the frequency to be analyzed was lower than this frequency, the data were downsampled to 250 Hz. Afterwards, continuous EEG signals were filtered *via* a 0.5–50 Hz bandpass filter. The raw data were processed with an automatic channel rejection method, and any removed channels were subsequently interpolated using spherical splines. Power line fluctuations at 50 Hz were then removed *via* the Cleanline EEGLAB plug-in (Mullen 2012). The data underwent artifact correction using the clean_rawdata function in EEGLAB, with a burst criterion set to 20 standard deviations. The data were re-referenced to an average reference computed using the average signal at all EEG electrodes. The EEG signal was then decomposed into 65 independent components *via* the infomax independent component analysis function included in EEGLAB, and each component was assigned its probability of being the signal source by the IClabel function (Pion-Tonachini *et al.* 2019). We used these labels to remove signals with a probability of 80% or greater of being myoelectric, eye movement, cardiac movement, line noise, or channel noise signals, which are all recognized as noise.

### Heartbeat-evoked potentials

The HEP is an event-related potential time-locked to the R peak and is considered to reflect the central cardiac activity process. The R peaks were used to segment the preprocessed EEG signals from −200 ms to 600 ms epochs. The inclusion criteria for the HEP analysis were participants who submitted ten or more reports of MW, ensuring a substantial amount of data to facilitate the reliable calculation of the HEP. As a result, six participants who reported fewer than ten instances of MW were excluded from the analysis.

Importantly, we did not conduct traditional subtraction-based baseline correction to avoid the theoretical issues. In HEP analysis, the baseline interval contains various systematic artifacts, including P-waves, Q-waves, and associated drift components. Therefore, conventional baseline correction methods, which assume random noise, may be inappropriate for calculating a baseline state of brain activity that is not tied to specific events.

Instead, we adopted a regression-based approach to statistically control for trial-by-trial variability in the baseline period for each channel (Alday 2019). We first defined a baseline window as −200 ms to −75 ms, which explicitly avoids the R-peak artifact. We then applied a General linear model to the data at each time point, using the mean voltage from this window, as well as its interaction with the experimental condition, as covariates. All subsequent HEP analyses were performed on the residualized waveforms, which were generated by removing the variance explained by these covariates from the original waveforms. This approach allows us to effectively remove linear influences of pre-stimulus variability (including potential contamination from physiological variability or other drifts) without artificially distorting the post-stimulus waveform.

To compare HEP amplitudes across conditions (e.g*.* attentional states, respiratory phases) without *a priori* assumptions about the timing or topography of the effects, we employed a nonparametric cluster-based permutation test across all channels and the entire epoch (0–600 ms) using the FieldTrip toolbox (Oostenveld *et al.* 2011). This data-driven method robustly controls the familywise error rate associated with multiple comparisons (Maris and Oostenveld 2007). For each comparison, dependent-samples t tests were first performed for every channel at every time point. Adjacent data points exceeding a threshold of *P* < .05 were grouped into clusters. The t-values within each cluster were summed to produce a cluster-level statistic. The significance of each observed cluster was then assessed by comparing its statistic to a null distribution of maximum cluster statistics, which was generated by randomly permuting the condition labels 1000 times. A cluster was considered significant if its *P*-value was ˂.025 (two-sided test) (Maris and Oostenveld 2007).

### Time–frequency analysis

To address our two purposes—to determine the differences between attentional states and the dynamics of the transition to awareness—we performed a time-frequency analysis using a Morlet wavelet transform, which was applied separately to time windows optimized for each one. The general parameters for the transform were consistent for both analyses: spectral power was calculated from 2 to 40 Hz, with the number of cycles increasing linearly from 3 as a function of frequency.

First, to compare brain activity across the different attentional states (focus, MW, and aware), we averaged the spectral power across the time dimension. This approach is based on the assumption that each epoch represents a single, stable cognitive state, yielding a single power value per channel and frequency for each trial. To identify significant differences in this time-averaged spectral power, we applied the same cluster-based permutation test procedure described in the HEP analysis. The test was performed across all channels and frequencies ranging from 2 to 40 Hz. Specifically, we conducted paired comparisons for focus versus MW and MW versus aware within each task condition (BF and SF). We also compared the task conditions (BF versus SF) within the focus and MW states, although these analyses revealed no significant differences.

In contrast, for the analysis detailed in the ‘Time series analysis via a statistical model’ section, the goal was to examine the temporal dynamics related to the transition to awareness of MW occurrence. For this purpose, the time-frequency data were reanalyzed using a continuous 10-s window before MW was reported, rather than the discrete time windows of the attentional states. For this second analysis, the full temporal resolution of the data was preserved, and this time course of spectral power was used as the input for the statistical model.

All the statistical analyses, excluding the cluster-based permutation tests, were conducted using the open-source software R (R 4.3.1). Most statistical analyses use linear mixed-effects models (LMMs) with the lme4 package (Bates *et al.* 2014). The specific fixed effects included in each model were tailored to the hypothesis under investigation. For instance, when the main effect of a single categorical factor, such as task condition (Figs 1D and 4A), attentional state (Fig. 1E and F), or timing (Fig. 4C), was assessed, the model included that factor as the sole fixed effect. Conversely, to investigate the influence of two factors and their potential interactions, a different model structure was used. This was the case for the analysis of RR intervals (Fig. 3A), the respiratory phase, and several analyses included in the Supplementary Material (e.g. examining time orientation or emotional valence; Supplementary Fig. 1), where models were constructed to include both main effects and their interaction terms. In all the models, we included a participant random intercept to account for individual differences. This approach ensured that each statistical test was appropriately specified for the particular question being addressed.

Statistical inference based on LMMs followed a multistep procedure. First, to assess the significance of the fixed effects, we performed a type III ANOVA on the LMM using the lmerTest package to obtain an F-statistic, with degrees of freedom estimated via the Satterthwaite method (Kuznetsova *et al.* 2017). Second, if a fixed effect was significant, we conducted post hoc pairwise comparisons between the levels of that effect using the emmeans package (Russell 2018). This procedure yielded t-statistics for each comparison, and *P*-values were adjusted for multiple comparisons using the Tukey method. Finally, we calculated standardized effect sizes, such as partial eta-squared (${\eta}_p^2$) and Cohen's d, from the model outputs.

### Time series analysis via a statistical model

#### Statistical modeling

We performed a time series analysis to identify the timing and temporal sequence of changes in the spectrum power in each frequency band. We constructed a time series model using a state–space model, with change point detection included to identify when EEG components shifted from the MW state to the aware state. We converted time units into time steps to perform the analysis. To enhance clarity, we reverted the time steps into actual time units in seconds after this analysis. Due to edge effects inherent in the wavelet transform, the window for the time-frequency analysis was shortened to span from 9.4 s to 0.5 s before the MW report.

The state–space model consists of two separate models: a system model and an observation model. The system model represents the true state, which cannot be measured directly, and the observation model represents the probabilistic distribution of the observed values. In this study, the spectrum power without noise is considered the true state, which is estimated from the observed values. We denote the true state as $\mu$ and the observed values as $Y$, with $n$ indicating the participants' number and $t$ symbolizing the time unit, which was converted from the actual time using the sampling frequency (Eq. 2).

Our focus was to identify when the spectrum power began to decrease, indicating a transition between attentional states. We define the $\mu$ parameter at time $t$ as $\mu$ at the previous time $t-1$ plus a trend component, $\delta$ (Eq. 3). Furthermore, we assumed that this $\delta$ is the same across individuals and varies randomly over time (Eq. 4). To model the trend's evolution over time, we set ${\delta}_{error_t}$ a parameter indicating the strength of the change to ${\sigma}_{\delta }$, which indicates the range of change. Assuming that trends in the time series change before and after a change point, we constructed two series of states, ${\mu}_{pre}$ and ${\mu}_{post}$, which started from the same value but evolved differently. That is, before the change point, $Y$ is generated according to ${\mu}_{pre}$, and after the change point, *Y* is generated according to ${\mu}_{post}$. The log likelihood is then estimated for the change points at each time point, and the point with the highest value is estimated as the point at which the trend changes.


(2) \begin{equation*} {\displaystyle \begin{array}{c}\left\{\begin{array}{@{}l}{Y}_{n,t}\sim Normal\left({\mu}_{n,t}^{pre},{\sigma}_n\right)\ if\ t< change\ point\\{}{Y}_{n,t}\sim Normal\left({\mu}_{n,t}^{post},{\sigma}_n\right)\ if\ t\ge change\ point\end{array}\right.\end{array}} \end{equation*}

(3) \begin{equation*} {\displaystyle \begin{array}{c}\left\{\begin{array}{@{}l}{\mu}_{n,t}^{pre}={\mu}_{n,t-1}^{pre}+{\sigma}_{n,t-1}^{pre}\\{}{\mu}_{n,t}^{post}={\mu}_{n,t-1}^{post}+{\sigma}_{n,t-1}^{post}\end{array}\right.\end{array}} \end{equation*}

(3) \begin{equation*} {\displaystyle \begin{array}{c}\left\{\begin{array}{@{}l}{\delta}_t^{pre}={\delta}_{t-1}^{pre}+{\sigma}_{\delta^{pre}}\ast{\delta}_{error_t}^{pre}\\{}{\delta}_t^{post}={\delta}_{t-1}^{post}+{\sigma}_{\delta^{post}}\ast{\delta}_{error_t}^{post}\end{array}\right.\end{array}} \end{equation*}

While acknowledging that the mean spectrum power and its noise component can differ among participants, we assume a shared change trend across individuals. This shared trend underpins our analysis, enabling us to study the overarching pattern of spectrum power changes, irrespective of individual variances. In particular, we considered a half-Cauchy distribution for the observed noise, and the distributions of the parameters (${\sigma}_{Y_n}$ and ${\sigma}_{\delta }$) were used to represent the time evolution of the trend. The half-Cauchy distribution represents the right side of the two symmetric halves of the Cauchy distribution and is recommended as a prior distribution of variance, especially for estimations performed with small samples (Gelman 2006; Polson and Scott 2012).


(4) \begin{equation*} {\displaystyle \begin{array}{c}\left\{\begin{array}{@{}l}{\delta}_{error_t}^{pre}\sim Normal\left(0,1\right)\\{}{\delta}_{error_t}^{post}\sim Normal\left(0,1\right)\end{array}\right.\end{array}} \end{equation*}

(5) \begin{equation*} {\displaystyle \begin{array}{c}{\sigma}_{Y_n}\sim Cauchy\left(0,5\right)\end{array}} \end{equation*}

(6) \begin{equation*} {\displaystyle \begin{array}{c}\left\{\begin{array}{@{}l}{\sigma}_{\delta^{pre}}\sim Cauchy\left(0,2.5\right)\\{}{\sigma}_{\delta^{post}}\sim Cauchy\left(\mathrm{0,2.5}\right)\end{array}\right.\end{array}} \end{equation*}

**Figure 2:**
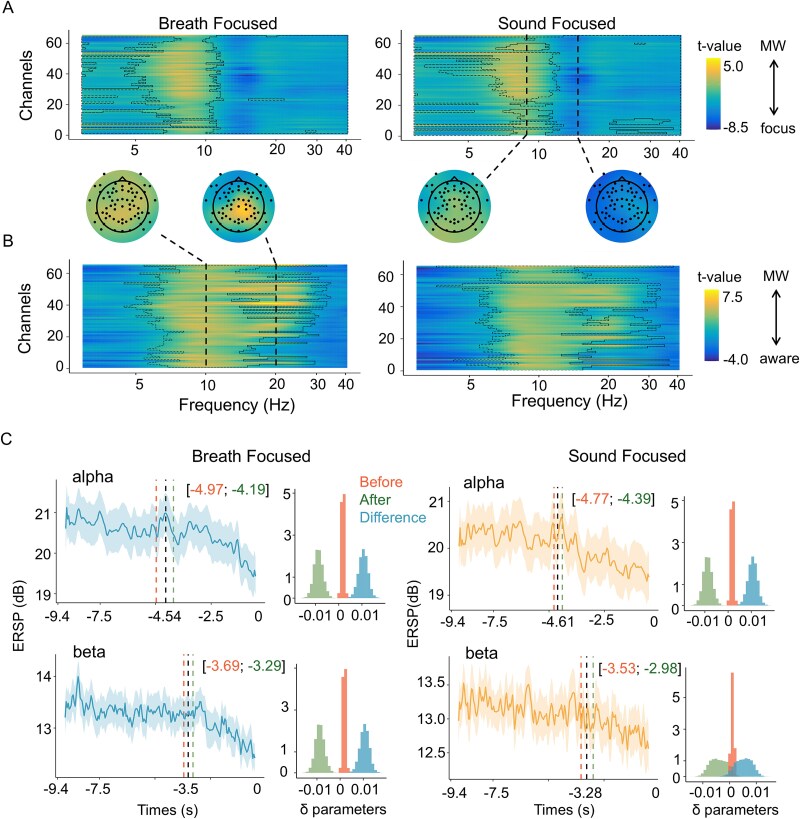
Changes in time–frequency components related to the transition to awareness of MW. (A) Difference in spectral power between the MW and focus states: We show the statistical value of the difference between the frequency of each electrode in the MW and focus states. The vertical axis shows the electrode number, and the horizontal axis shows the frequency band. Warmer colors indicate greater intensity in certain frequency bands during MW, whereas cooler colors indicate greater intensity in the focus state. The dotted lines mark the areas that reflected the significant difference. (B) Difference in spectral power between the MW and aware states: Same as in (A), except for the state being compared. Warmer and cooler colors indicate increased intensity in the MW and aware states, respectively. (C) Time series of the spectrum power before MW is reported: The horizontal axis represents the time in seconds from when MW is reported, and the vertical axis represents the spectral power values. The bold line represents the mean value, and the range represents the standard error. The vertical dotted line represents the 95% credible interval (CI) of the estimated change point. The interval before the estimated change point is the segment before the red line, and the interval after the change point is on the right side of the green line. The trends before and after the change point are plotted on the right side of the time series. The red and green histograms show the distributions of the trend parameters before and after the change point, respectively. The difference parameter indicates the value obtained by subtracting the trend after the change point from the value before the change point in the blue histogram.

For the time series modeling, we selected a single representative electrode. An initial cluster-based permutation test across all channels revealed that the decreases in alpha and beta power were not localized but were distributed across a widespread central-parietal cluster (see Fig. 2). Given the high computational demands of the time series model and because of this widespread effect, we selected the Cz electrode as a representative channel because it was located within the central-parietal cluster that reflected the significant effect reported below, and is free from both hemispheric (left–right) and anterior–posterior bias due to its central position on the scalp.

### Model implementation

The statistical model was estimated via the Bayesian approach using R and the R package Rstan (Stan Development Team 2024). We employed the Markov chain Monte Carlo (MCMC) method for parameter estimation. In the MCMC method, parameters are estimated by accumulating numerous samples from the posterior distributions. We obtained 6000 simulated samples from the posterior distribution for each parameter. The simulated samples were preceded by 1000 burn-in samples, which were discarded from the analysis. The burn-in samples were discarded to exclude the effects of the initial values. The MCMC chain was thinned by including only every second draw to reduce temporal autocorrelations among the samples, yielding 2500 simulated posterior observations. We conducted four samplings and obtained 10 000 simulation data points for each parameter.

The convergence of the MCMC algorithm across the four chains was assessed for each parameter using $\hat{R}$ as the index of the variance between the chains relative to the variance within a chain (Gelman *et al.* 2013). If the MCMC algorithm converges across all chains, $\hat{R}$ is close to 1.0. To estimate the convergence of the algorithm, we set the criterion to a value ˂1.1. In summary, across all the parameters, $\hat{R}$ values of ~1.0 indicated convergence across the four chains. In addition, we calculated the effective sample size (ESS) for each parameter to assess the sampling efficiency. Across all key parameters, the ESS was well above 1000, indicating efficient exploration of the posterior distribution. The combination of $\hat{R}$ values near 1.0 and high ESS values confirmed that the MCMC algorithm successfully converged for all the parameters in the present model.

## Results

### Subjective experiences and task performance

In the present study, the average number of MW reports was 28.39 (*SD* = 19.22; *range* = 3–96) in the BF condition and 31.70 (*SD* = 20.51; *range* = 4–90) in the SF condition (Fig. 1D). Although there was a pattern of slightly more reports under the SF condition, a comparison based on our LMMs revealed no statistically significant difference in the number of reports for the two conditions (*t*(43) = −1.88, *P* = .067, *d* = −0.57). First, task performance was analyzed to confirm that the MW reports obtained in this study showed the same trends as those reported in previous studies did. We compared the reaction time (RT) and RTV. RTV was calculated by dividing the standard deviation of RTs within each state by that state's mean RT. Although the RTs did not significantly differ between the attentional states (*F*(2, 86) = 2.912, *P* = .060, ${\eta}_p^2$ = .06), a significant difference was found for the RTV (*F*(2, 86) = 8.365, *P* < .001, ${\eta}_p^2$ = .16). Subsequent analyses revealed less variability during the focus state than during the MW and aware states (MW: *t*(86) = 2.837, *P* = .016, *d* = .62; aware: *t*(86) = 3.970, *P* < .001, *d* = .86; Fig. 1E and F). No significant difference in variability was found between the MW and aware states (*t*(86) = −0.021, *P* = .505, *d* = −.004). Consistent with previous research (Seli *et al.* 2013; Shinagawa *et al.* 2023), our study revealed significant variability in the responses in the MW state.

### Changes in whole-brain activity during the transition from MW to awareness of its occurrence

Next, we clarified how the brain dynamics were characterized during the transition from the MW state to the aware state. Using a cluster-based permutation test, we compared the spectral power across various frequencies at each electrode between the MW and focus states (Fig. 2A). Assuming a consistent attentional state within this interval, we averaged the data over time, which yielded values for each channel by frequency. In Fig. 2A, warmer colors denote higher intensity in certain frequency bands during MW, whereas cooler colors indicate greater intensity in the focus state. The dotted lines indicate areas identified as significant in the cluster analysis (*P* <. 025). Our analysis revealed a uniform pattern across both task conditions: theta (below 7 Hz), beta (13–30 Hz), and gamma (above 30 Hz) wave activities were higher in the focus state than in the MW state (BF: *cluster statistic* = −3 516, *P* < .001; SF: *cluster statistic* = −3 769, *P* < .001). The frequency maps of the MW states, compared to those of the focus state, are similar to those of previous studies (Kam *et al.* 2022), supporting the validity of the MW states identified in the present study.

Additionally, we analyzed the difference between the frequency in the MW state and that in the aware state, and the results are presented in Fig. 2B. This figure shows the differences in intensity for each frequency band and channel, with warmer and cooler colors indicating increased intensity in the MW and aware states, respectively (Fig. 2B). The results demonstrated that alpha and beta band activities were lower in the aware state than in the MW state (BF: *cluster statistic* = 2 162, *P* = .003; SF: *cluster statistic*, *P* = .006). Taken together, these analyses reveal a pattern of spectral power changes across the attentional states. We observed a gradual decrease in beta power from the focus state to the MW state and further to the aware state. For alpha power, a significant decrease was specifically observed in the transition from the MW state to the aware state.

On the basis of the cluster-based permutation test, we identified when alterations in alpha and beta waves began and clarified the sequential relationship between these changes using time series analysis with change point detection. The time series analysis of the Cz electrode data revealed a sequential relationship between these changes (Fig. 2C). For the alpha wave, the analysis highlighted a 95% credible interval (CI) for the change points, which indicates the starting point of a decreasing trend, ranging from 4.97 to 4.19 s before the MW reports in the BF condition. These results suggest a 95% probability that a change in the time series occurred within this interval. The estimated mean change point was 4.67 s before MW was reported (SF: mean = 4.61, 95% CI = [4.39; 4.77]). We plotted the trend parameters before and after the change points on the right side of the time series in Fig. 2C. A comparison of the mean values of the trends before and after the change point revealed that the lower limit of the 95% CI exceeded 0, indicating that alpha wave activity tended to decrease after the change point (BF: mean = 0.00984, 95% CI = [0.00592; 0.0132]; SF: mean = 0.152, 95% CI = [0.0104; 0.0192]). Similarly, the change point for beta wave activity was estimated to occur 3.69–3.29 s before MW was reported with a 95% probability (BF: mean = 3.51 s before the report; SF: mean = 3.28, 95% CI = [2.98; 3.53]). A comparison of the mean values of the trends before and after the change point revealed that the lower limit of the 95% CI exceeded 0, indicating that beta wave activity decreased after the change point (BF: mean = 0.0105, 95% CI = [0.00717; 0.0137]; SF: mean = 0.005, 95% CI = [0.0002; 0.0101]). The results of the change point analysis suggest that a decrease in beta wave activity typically occurs after a decrease in alpha wave activity.

### Changes in physiological states and their central process in the transition from MW to awareness of its occurrence

#### Interbeat interval as an index of physiological states

**Figure 3:**
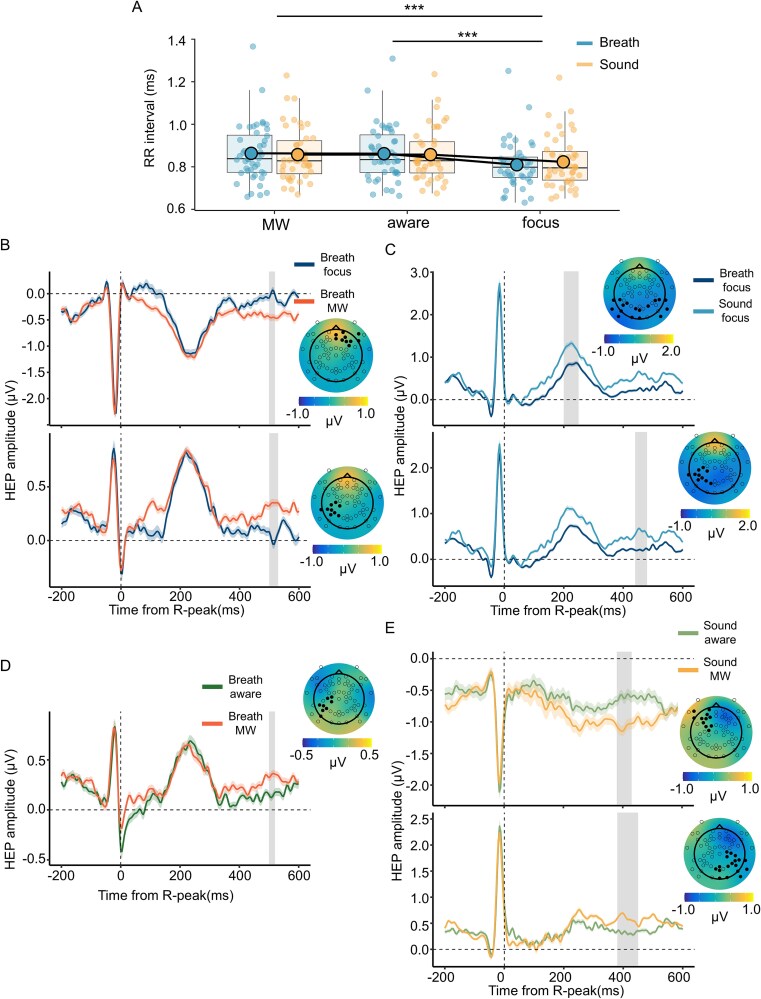
Cardiac activity and its central process in each task condition and attentional state. (A) Cardiac activity in each task condition and attentional state: The horizontal axis shows the attentional state, the vertical axis shows the RR interval, and the color indicates the task condition. Each dot indicates a participant. (B)–(E) Modulation of HEP by attentional state and task condition: Grand average HEP waveforms for the two compared conditions, averaged across all channels contributing to the significant effect identified by the cluster-based permutation test. The vertical axis shows the amplitudes, and the horizontal axis shows the time from the R peak. The bold line indicates the mean amplitude, and the shaded range represents the standard error. The gray area on the time axis highlights the significant difference in the time window. We also displayed the topography map of the difference in voltage between the two conditions, which was calculated by subtracting the condition listed second in the legend from that listed first (e.g*.* for B, focus—MW), averaged across the corresponding time window. All electrode positions are indicated by white circles, whereas electrodes contributing to the significant effect are highlighted with solid black circles. The compared conditions are as follows: (B) the focus and MW states within the BF condition; (C) the focus state in the BF condition and that in the SF condition; (D) the aware and MW states within the BF condition; and (E) the aware and MW states within the SF condition. Where multiple significant effects (clusters) were identified in a single comparison (panels B, C, E), they are plotted separately (e.g. upper panel: Frontal/earlier clusters; lower panel: Posterior/later clusters) for clarity. ^***^P < .001. Note: A small, consistent visual offset between the R-peak artifact (on the EEG waveform) and t = 0 is present. This processing offset does not reflect R-peak misidentification and does not affect the statistical comparisons between conditions.

To explore the physiological impact of arousal-related brain activity on the physiological state, we analyzed how interbeat intervals vary with attentional states (RR interval; Fig. 3A). LMMs on the RR intervals revealed a significant main effect of attentional state (*F*(2, 212.02) = 29.915, *P* < .001, ${\eta}_p^2$=.22) but no significant main effect of task condition (*F*(1, 212.23) = 0.291, *P* = .590, ${\eta}_p^2$=.0013) and no significant interaction between the two factors (*F*(2, 212.02) = 1.4073, *P* = .247, ${\eta}_p^2$=.001). Subsequent analysis revealed that participants’ heart rates were faster in the focus state than in the MW and aware states under each condition (MW: *t*(212) = 6.762, *P* < .001, *d* = .93; aware: *t*(212) = 6.634, *P* < .001, *d* = .91; Fig. 3C). No significant differences between the participant heart rate in the MW state and that in the aware state (*t*(212) = 0.128, *P* = .991, *d* = .02) were found. A previous study revealed that MW was associated with low arousal to external stimuli during response tasks (Unsworth and Robison 2018).

#### Heartbeat-evoked potentials as indices of the central process of cardiac activity

To investigate how the central cardiac signal process is modulated by the attentional state, we compared the HEP using a whole-scalp, cluster-based permutation test (Fig. 3B–E). First, we compared the focus state to the MW state within the BF condition, which revealed a significant HEP modulation. This effect was reflected in two clusters: a positive effect over frontal electrodes (*cluster statistic* = 32.36, *P* = .023; ~500–520 ms) and a negative effect over parietal electrodes (*cluster statistic* = −97.21, *P* = .018; ~500–530 ms) (Fig. 3B). These *P*-values refer to the probability of observing such cluster-level statistics under the null hypothesis, not to the local significance of specific electrodes or time points. Second, we compared the focus state to the MW state within the SF condition. In contrast, this comparison did not reach significance. These results suggest that when the participants focused on their breath, the later HEP amplitudes were greater at the front and smaller at the parietal electrodes than during the MW state.

To examine the differential effects of internal *versus* external attentional focus, we compared the focus state in the BF condition to that in the SF condition. This comparison also revealed a significant difference. This effect was reflected in two negative clusters over the parietal electrodes: one in the earlier time window (*cluster statistic* = −175.48, *P* = .009; ~200–250 ms) and another in the later time window (*cluster statistic* = −200.46, *P* < .001; ~440–480 ms), indicating that the HEP amplitudes were smaller in the BF condition (Fig. 3C). These results revealed that the central cardiac signal process is significantly modulated when attention is directed toward an internal bodily signal, a modulation characterized by suppression of the parietal later component.

Next, to address our primary question regarding the transition from MW to awareness of its occurrence, we directly compared the HEP in the aware state to those in the MW state. We performed this comparison separately for both the BF and SF conditions (Fig. 3D and E). In the BF condition, the comparison revealed a significant modulation. This effect was reflected by a negative cluster at parietal electrodes (*cluster statistic* = −27.53, *P* = .021; ~500–520 ms) (Fig. 3D). Also, the comparison within the SF condition revealed significant modulation. This effect was characterized by two clusters: a positive effect over frontal electrodes (*cluster statistic* = 195.99, *P* = .023; ~380–430 ms) and a negative effect over parietal electrodes (cluster statistic = −449.01, *P* < .001; ~380–450 ms) (Fig. 3E). We observed a consistent suppression of the parietal later component in the Aware state relative to the MW state across both task conditions. This pattern, which indicates an enhancement of the central cardiac process, is consistent with the similar enhancement observed during the focus state in the BF condition.

#### Relationships among respiratory phases, cardiac activity, and central processes during the transition from MW to awareness of its occurrence

**Figure 4:**
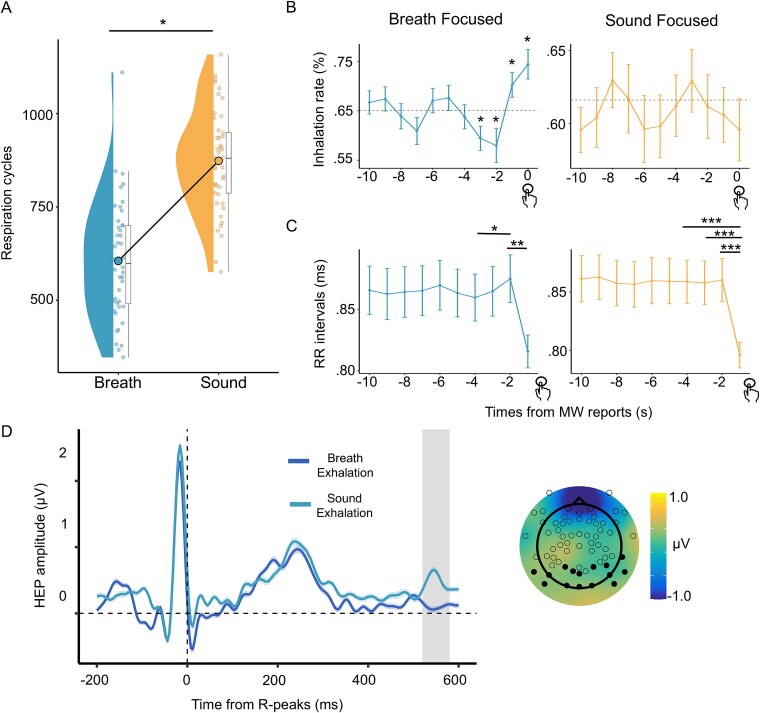
Changes in respiration and the accompanying RR interval and its central process before MW was reported. (A) Number of respiratory cycles during the task: The task condition is shown on the horizontal axis, and the number of cycles is shown on the vertical axis. Each dot represents a participant. (B) Percentage of the inhalation phase each second before MW was reported: Inhalation rates are shown on the vertical axis, with the time in seconds (s) from when MW was reported shown on the horizontal axis. The horizontal dotted lines indicate the baseline inhalation rate calculated *via* random sampling from the entire dataset for each condition. The error bars indicate the standard errors. (C) RR interval at each second before MW was reported: The RR interval is shown on the vertical axis, with the time in seconds from the reports of MW on the horizontal axis. The error bars indicate the standard errors. (D) Modulation of HEP waveforms during the exhalation phase: This panel compares the HEP recorded during the exhalation phase in the BF conditions to those in the SF condition. The figure format is identical to that used in Fig. 3. ^*^P < .05, ^**^P < .01, ^***^P < .001.

In our final analysis of the transition to awareness of MW, the respiratory phases and their relationships with cardiac activity and HEP before MW was reported were examined (Fig. 4). First, we revealed that the respiratory cycle decreased in the BF condition, suggesting slower respiration than in the SF condition (*t*(43) = −11.105, *P* < .001, *d* = 3.39; Fig. 4A). Next, we examined whether a specific pattern of respiratory phases corresponds to the spontaneous awareness of MW. For this purpose, we used a one-sample t test to determine whether the proportion of the inhalation phase at each second prior to MW reports significantly differed from the chance-level baseline. This baseline was calculated as the mean proportion derived from 10 000 random samples drawn from the entire respiratory time series data. Since every second during the task was classified as belonging to a specific respiratory phase (inhalation or exhalation), an increase in the rate of one phase at any given time implies a decrease in the rate of the other phase at that same time point. The results revealed a significant decrease in the inhalation rate compared with the baseline 2 and 3 s before MW was reported only in the BF condition (2 s: *t*(43) = −2.070, *P* = .044, *d* = −.63; 3 s: *t*(43) = −2.318, *P* = .025, *d* = −.71; Fig. 4B). Remarkably, as the moment of awareness approached, the participants were more likely to be in the inhalation phase (0 s: *t*(43) = 3.134, *P* = .003, *d* = .96; 1 s: *t*(43) = 2.046, *P* = .046, *d* = .62). These patterns were not observed in the SF condition.

To investigate how cardiac activity varies with the respiratory phase, we analyzed changes in the RR interval over time during periods in which the respiratory phase changed. For this purpose, the RR interval between each second was labeled with the later second—for example, heartbeats that occurred at ˂1 s, between 1 and 2 s, and between 2 and 3 s before MW was reported were labeled 1 s, 2 s, and 3 s, respectively. To evaluate the RR interval based on the respiratory phase results, the RR intervals were labeled on the basis of time rather than on the basis of the cardiac beat. Through this method, the impact of the respiratory phase at each second is reflected in the RR interval for that second; that is, if multiple heartbeats occurred within 1–2 s, both were labeled 1 s. We analyzed the RR intervals that occurred within 4 s before MW was reported in each condition via LMMs, with the timing of the RR interval considered a fixed effect and the participant information considered random effects.

The analysis revealed a significant main effect of the timing of the RR interval (*F*(3, 117.95) = 5.3162, *P* = .002, ${\eta}_p^2$ = .12; Fig. 4C), with subsequent analyses indicating that the RR interval was longer at 2 s than at 4 s before MW was reported (*t*(118) = 2.817, *P* = .029, *d* = 0.52). The RR interval was shorter at 1 s before MW was reported (*t*(118) = −3.785, *P* = .001, *d* = −.70). This pattern suggests a decrease in heart rate in the exhalation phase, the rate of which increased 3 s before MW was reported, followed by an increase in heart rate in the inhalation phase, the rate of which increased 1 s before MW was reported. Similarly, in the SF condition, the main effect of timing was significant (*F*(3, 117.92) = 9.3048, *P* = .002, ${\eta}_p^2$ = .19; Fig. 4C), with comparisons between timings revealing a shorter RR interval 1 s before MW was reported than at 2–4 s (2 s: *t*(118) = 4.651, *P* < .001, *d* = 0.86; 3 s: *t*(118) = 4.254, *P* < .001, *d* = 0.78; 4 s: *t*(118) = 4.412, *P* < .001, *d* = 0.81), indicating an increase in heart rate before MW was reported.

In addition, the HEP was compared for heartbeats occurring at 0–2 s (inhalation) and 2–4 s (exhalation) before MW was reported to test whether brain activity was associated with respiratory and cardiac signals (Fig. 4D and E). This analysis is preliminary because only half the time window is used compared with the analysis of attentional states, reducing the number of trials. Additionally, we note that the respiratory phase labels were determined on the basis of which phase was increased relative to the baseline in the BF condition. To characterize the respiratory patterns preceding the awareness of MW, we labeled each interval as the respiratory phase, although no changes were observed in the SF condition. When only heartbeat data were used for analysis in each phase, the number of trials used to calculate the HEP halved again. We labeled the data to ensure a larger number of trials and that the analysis was the same as that performed in the RR interval. During the exhalation phase, the comparison between the BF and SF conditions revealed a significant difference (*P* = .020). This effect was reflected by a negative cluster over parietal electrodes (*cluster statistic* = −98.97; ~520–580 ms), indicating that HEP amplitudes were greater in the SF condition than in the BF condition (Fig. 4E). In contrast, no significant differences were found for the same comparison during the inhalation phase or when the respiratory phases were compared within each task.

## Discussion

We examined the dynamics of brain activity, the physiological state, and the central process associated with the transition from the MW state to the awareness of its occurrence. The results revealed that the HEP amplitudes were suppressed when the participants focused on their respiration than when they were in both the MW state and the sound-focused state (Fig. 3B and C). Moreover, the HEP amplitudes similarly suppressed when the participants became aware of MW than when they were in the MW state (Fig. 3E and F), implying that this transition is characterized by the enhancement of the central nervous system processes of cardiac activity. Additionally, changes in respiratory phases and cardiac activity before MW reports were observed when the participants were asked to focus on respiration (Fig. 4A). We also observed that changes in the respiratory phase before the MW report were associated with changes in cardiac activity and its central process (Fig. 4C and E). While our correlational findings cannot establish causality, they provide a basis for a working hypothesis about the underlying mechanisms. In the following sections, we discuss the implications of our key findings in this context.

The HEP is recognized as the central process of cardiac activity (Schandry *et al.* 1986; Coll *et al.* 2021). Recent studies have revealed that HEP is altered by the attentional resources devoted to processing the internal body state, including respiration (Zaccaro *et al.* 2022; Tanaka *et al.* 2023). Since attention is not necessarily allocated to the body during MW, it is reasonable that the HEP amplitudes modulated when attention was focused only on respiration (Fig. 3C and D). These results suggest that the increased HEP amplitudes during the transition to awareness of MW may indicate increased central cardiac activity processes and attentional resources directed toward internal states (Fig. 3E and F). Many studies have demonstrated a link between the central cardiac activity process and conscious experience (Park and Tallon-Baudry 2014; Salomon *et al.* 2016; Candia-Rivera *et al.* 2021). In particular, an MEG study revealed that the self-relatedness of spontaneous thoughts covaried with heartbeat-evoked responses, indicating that cardiac reactivity is modulated by a first-person perspective (Babo-Rebelo *et al.* 2016). These results suggest that the constant neural updating of the visceral states forms a first-person perspective for conscious experiences (Tallon-Baudry *et al.* 2018). The HEP changes observed in this study may support the hypothesis that enhanced central cardiac processing accompanies shifts in conscious experience.

We next discuss a potential explanation for the relationship between awareness of MW and enhanced cardiac processing from the perspective of interoception, which is the process by which the nervous system senses and integrates information originating from the body (Khalsa *et al.* 2018). A previous study on prospective memory—the memory of future intentions—reported that cue recognition accelerates cardiac activity to facilitate the execution of intended actions (Umeda *et al.* 2016). Furthermore, interoceptive accuracy (IAcc)—the ability to accurately detect one’s internal state—mediates performance in prospective memory tasks (Umeda *et al.* 2016). These results suggest that accurate perception of the physiological changes caused by cues enhances the processing of the underlying cause and the retrieval of the intended action. In self-caught methods, participants must report their MW, a process that inherently involves prospective memory. The heart rate increase shortly before MW is reported (Fig. 4C) may reflect an orienting or readiness response for subsequent actions. In addition, both our study and the previous study revealed that various autonomic activities occur before MW is reported (Fig. 4B and C; Munn *et al.* 2021). Accurate perception of these fluctuations may promote the processing of relevant cues, driving awareness of the occurrence of MW. In addition, increased activation in the dorsal anterior cingulate cortex and anterior insula—regions implicated in interoception—just prior to reports of MW support a role in the detection of internal changes (Menon and Uddin 2010; Hasenkamp and Barsalou 2012; Uddin 2015). Therefore, we suggest that not only changes in the central cardiac activity process but also the perception of these changes contribute to the detection of MW.

Our findings revealed a distinct respiratory pattern specific to the BF condition, where participants tended to be in the exhalation phase before becoming aware of MW and in the inhalation phase when reporting MW (Fig. 4B). We propose a working hypothesis that attention to breathing modulates cardiorespiratory coupling, thereby modulating the central cardiac signal process. First, attention to the respiratory system led to a slower respiratory cycle in the BF condition than in the SF condition (Fig. 4A), which is consistent with findings that meditation practice leads to a slower respiratory cycle (Wielgosz *et al.* 2016). During slow breathing, cardiorespiratory coupling, such as respiratory sinus arrhythmia (RSA), is strongest (Hirsch and Bishop 1981), which leads to more pronounced cardiac dynamics (Eckberg 1983). This enhanced coupling may explain our findings in the BF condition, namely, the lower heart rate and higher HEP amplitude during the exhalation-dominant period (Fig. 4C and E). The finding that the central cardiac activity process increases during the exhalation phase is also consistent with prior research (Zaccaro *et al.* 2022). In contrast, this attentional modulation was not observed in the SF condition, which explains the absence of a systematic respiratory pattern preceding awareness in that context. In summary, our results suggest that intentional attention to respiration slows breathing, which may in turn enhance cardiorespiratory coupling and amplify the central cardiac signal process, thereby facilitating the transition to the awareness of MW.

The time series analysis revealed a decrease in alpha waves followed by a reduction in beta waves before MW was reported (Fig. 2C). Alpha waves are associated with internally focused states and MW (Kam *et al.* 2022; Fig. 2A). The timing of the observed decrease in the alpha waves (4–5 s prior to the report) aligns with previous findings showing that arousal-related neural activity increases 3.5–5 s before MW reports (Munn *et al.* 2021; Shinagawa *et al.* 2021), supporting our interpretation that this change reflects the transition from MW. Beta waves, which are linked to attention control, cognitive load, and stress (Attar 2022; Díaz *et al.*, 2019), decreased after the departure from MW, suggesting that they reflect cognitive load rather than internal thought regulation. In further support of this interpretation, beta power increases during states of lower parasympathetic activity (Karjalainen *et al.* 2024), and we observed an increase in RR intervals ~2 s after the beta band decreased in the BF condition (Fig. 4C). These patterns are consistent with models suggesting that the continuous allocation of limited cognitive resources to a task leads to potential opportunity costs, which may become aversive, prompting a transition to alternative mental or behavioral states (Kurzban *et al.* 2013; Struk *et al.* 2020). Indeed, MW frequency varies with task motivation (Seli *et al.* 2015; Robison *et al.* 2020; He *et al.* 2023), and being alone with one’s thoughts can be so unpleasant that individuals may prefer aversive stimulation (Wilson *et al.* 2014). A simulation study qualitatively reproduced this process (Shinagawa and Yamada 2025), suggesting that continued engagement in the same internal or external state facilitates transitions to other thought states. The observed decrease in cognitive load and parasympathetic nervous system activity during the transition from MW is consistent with predictions derived from this theoretical framework and provides empirical support for its validity. The physiological changes accompanying these shifts may underlie changes in the central bodily signal process that influence awareness of MW.

In interpreting our findings, it is important to consider the potential confounding influence of motor-related activity. First, it could be argued that the observed neurophysiological changes between attentional states and task conditions simply reflect the motor response. Since motor responses were required across all attentional states (Fig. 1E and F), the observed differences are unlikely to be driven by the mere presence of responses. This reasoning, however, does not apply to direct comparisons between task conditions. The frequency of motor responses was intentionally lower in the BF condition than in the SF condition to maintain participant engagement. We therefore acknowledge that this difference in motor frequency is a limitation for result interpretation based on direct between-condition comparisons, such as the number of MW reports or respiratory cycles.

In addition to these motor response presence and frequency factors, a more specific alternative interpretation concerns the preparation for the voluntary MW report itself, including the readiness potential and its relationship with the respiratory cycle. This interpretation is based on prior work showing that voluntary movements are more likely to be initiated during the exhalation phase (Park *et al.* 2022). However, this simple motor preparation account is inconsistent with three key aspects of our data. The most definitive evidence is that voluntary actions in the literature are initiated during exhalation, whereas our participants made their reports of MW during inhalation (Fig. 4B). Furthermore, the timing of our key neural findings (an alpha decrease of ˃4.5 s before the MW reports) precedes the typical onset of the readiness potential by ˃2 s (Schurger *et al.*, 2021). Additionally, we note that the distinct respiratory pattern observed in the BF condition was not present in the SF condition. Although motor components were consistently present in our paradigm, they cannot fully explain the pattern of our results, suggesting that our findings primarily reflect an awareness of MW occurrence that precedes the motor response of reporting.

The present study has several limitations. The primary limitation is that our correlational design cannot establish causality between the observed phenomena. Instead, on the basis of the observed associations, we can propose only working hypotheses regarding causal relationships. For instance, while we revealed a temporal relationship between changes in alpha and beta activity, it remains uncertain whether leaving the MW state causes a decrease in cognitive load. Similarly, while our working hypothesis suggests that increased processing of bodily signals drives the awareness of MW occurrence, reverse causality is also plausible; that is, the shift in attentional orientation to the self could trigger the observed increase in HEP amplitude.

In addition, the effects of signals from interoceptive modalities other than cardiac activity were not investigated. Recent research has revealed that the stomach may constrain spontaneous brain activity (Richter *et al.* 2017). Respiration, which was examined only in terms of phase changes in the present study, is also known to influence rhythmic brain activity in multiple frequency bands (Heck *et al.* 2022). Thus, we must consider not only the independent effect of each organ on brain activity but also the interactions between systems.

Additionally, we could not fully account for the complex coordination of basic bodily functions in explaining the changes in physiological indices before MW reports. In the present study, physiological indices were treated as independent factors in relation to the process of awareness. However, these systems are intricately linked through complex, coordinated processes, such as RSA, where cardiac activity fluctuates with the respiratory phase (Yasuma and Hayano 2004). This inherent coupling means that we cannot rule out the possibility that the observed changes in cardiac activity and its central process are attributable not only to the cognitive processes proposed in this study but also to these underlying physiological interactions. Consequently, while our correlational results are consistent with a working hypothesis in which changes in respiration modulate cardiorespiratory coupling, they do not preclude the possibility that these are merely concomitant effects without a direct causal link. In summary, future research should clarify the interactions between various interoceptive modalities, including their causal effects on awareness, while carefully considering the influence of these basic bodily functions.

A further limitation arises from our operational definition of MW. We defined MW conventionally as task-unrelated and stimulus-independent thought (Stawarczyk *et al.* 2011; Smallwood and Schooler 2015); however, recent work highlights that this category may contain subtypes with different dynamic properties, such as ruminative thought *versus* freely moving thought (Christoff *et al.* 2016). These subtypes may have distinct brain–body physiology (Ottaviani *et al.* 2013). Our methodology was not designed to distinguish them, as our primary focus was to characterize the transition to awareness rather than the phenomenology of the states themselves. Similarly, this limitation extends to the distinction from other forms of attentional disengagement, such as mind-blanking (MB). This is important, as recent studies suggest that MB has a distinct brain–body physiology from that of MW (Corcoran *et al.* 2025; Boulakis *et al.* 2025). While our study did not explicitly control MB, our task instructions—which required participants to report the occurrence of specific thoughts and their content—make it unlikely that content-free MB states were systematically included in our MW reports. Therefore, the findings of this study should be interpreted as specific to the conventionally defined MW state, and future research would benefit from methodologies that can separate these different forms of spontaneous thought and attentional disengagement.

In summary, we clarified how brain–body interactions are associated with the transition from MW to awareness of its occurrence *via* EEG and physiological indices. Our results revealed that the central cardiac activity process increased during this transition. Moreover, the shift away from MW may be accompanied by a reduction in cognitive load and related physiological changes, which may affect the brain’s processing of bodily states and facilitate the transition. Furthermore, when participants directed their attention to breathing, the respiratory cycle was altered, and the processing of bodily signals was amplified. However, the present study is limited to describing the neurophysiological dynamics associated with the transition to awareness of MW occurrence. Future research should aim to elucidate the mechanism by which these gradual, preparatory changes generate the instantaneous, subjective experience of becoming aware of MW. Clarifying these brain–body interactions will be crucial for our future understanding of how internal bodily signals shape moment-to-moment changes in conscious experience.

## Supplementary Material

new_sfig1_niaf059

Supplementary_Table_1_niaf059

Supplementary_Table_2_niaf059

Supplementary_Table_3_niaf059

Revised_supplementary_niaf059

## Data Availability

The data that support the findings of this study are available upon request from the corresponding author [K. S.]. The data are not publicly available because we did not obtain consent from the participants. The codes used to analyze the data are available on GitHub at https://github.com/shina-k/Neurosci.-Conscious.2025.
