# Supplementary figures and images for: Brain–body interactions associated with the transition from mind wandering to awareness of its occurrence
Source: Neurosci Conscious. 2025 Dec 15;2025(1):niaf059. doi: 10.1093/nc/niaf059 (PMC12704443; doi:10.1093/nc/niaf059)

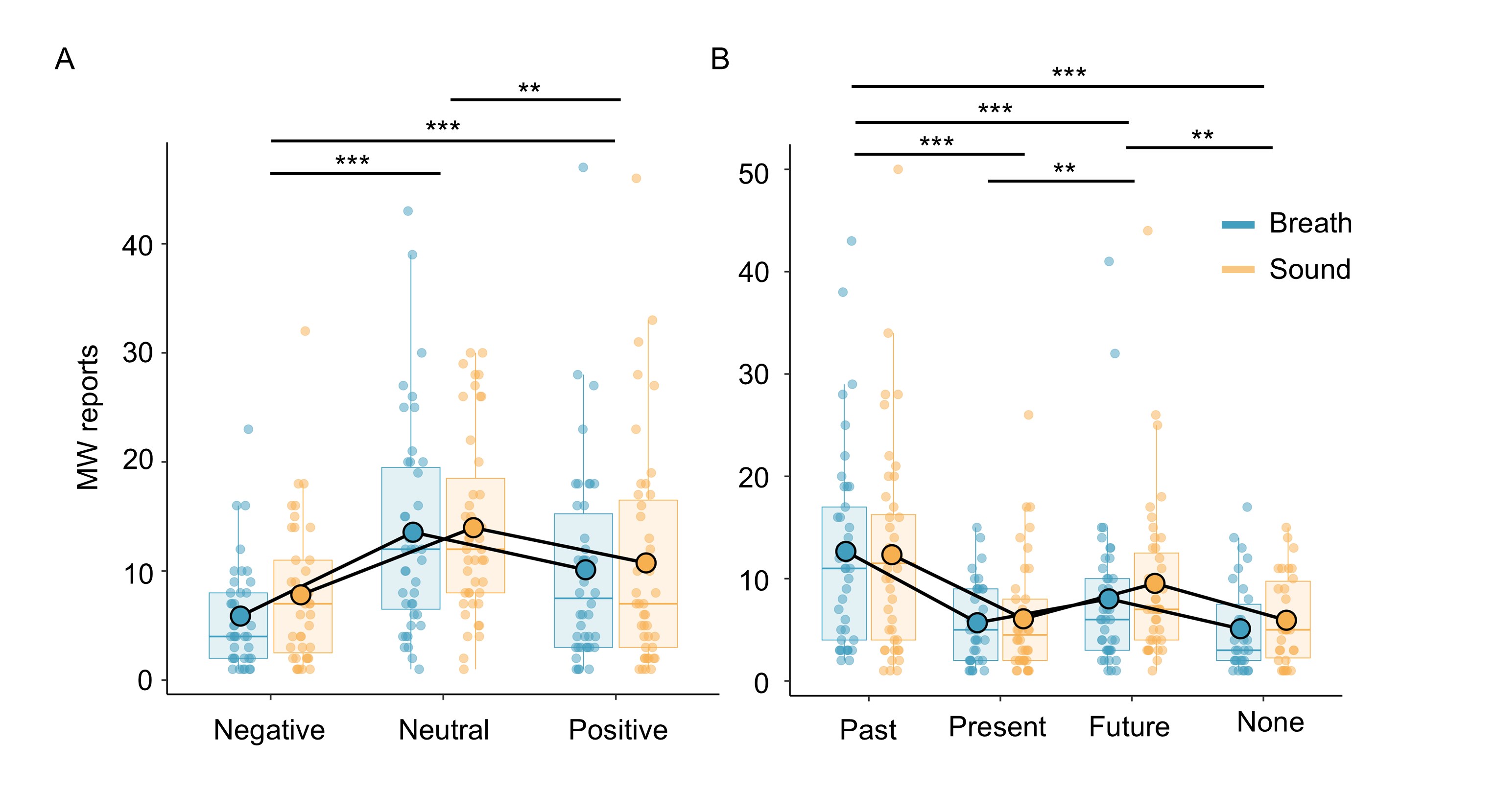

Supplement: new_sfig1_niaf059 [file new_sfig1_niaf059.jpeg]
