# Supplementary material for: Brain–body interactions associated with the transition from mind wandering to awareness of its occurrence
Source: Neurosci Conscious. 2025 Dec 15;2025(1):niaf059. doi: 10.1093/nc/niaf059 (PMC12704443; doi:10.1093/nc/niaf059)
Supplement: Supplementary_Table_1_niaf059 [file supplementary_table_1_niaf059.docx]

**Supplementary Table 1. Statistics on differences in the frequency of thought types**

| **Type** | **Between** | **df** | **t value** | **d** | **p value** |
| --- | --- | --- | --- | --- | --- |
| Emotion | Nega-Neutral | 202 | -7.483 | -1.05 | <.001 |
|  | Nega-Posi | 201 | -4.031 | -0.57 | <.001 |
|  | Neutral-Posi | 200 | 3.545 | 0.50 | .0014 |
| Time | Past-Now | 260 | 7.809 | 0.97 | <.001 |
|  | Past-Future | 260 | 4.230 | 0.52 | <.001 |
|  | Past-None | 263 | 7.157 | 0.88 | <.001 |
|  | Now-Future | 261 | -3.678 | -0.46 | .0016 |
|  | Now-None | 264 | 0.024 | 0.003 | 1.000 |
|  | Future-None | 264 | 3.391 | 0.42 | .0044 |
