# Supplementary material for: Brain–body interactions associated with the transition from mind wandering to awareness of its occurrence
Source: Neurosci Conscious. 2025 Dec 15;2025(1):niaf059. doi: 10.1093/nc/niaf059 (PMC12704443; doi:10.1093/nc/niaf059)
Supplement: Supplementary_Table_2_niaf059 [file supplementary_table_2_niaf059.docx]

**Supplementary Table 2. Statistics on the correlation between interoceptive accuracy and the frequency of thought types**

| **Type** | **Variable** | **Correlation** | **P value** |
| --- | --- | --- | --- |
| Emotion | Negative | .229 | .139 |
|  | Neutral | .0106 | .946 |
|  | Positive | -.146 | .351 |
| Time | Past | .004 | .981 |
|  | Now | .159 | .309 |
|  | Future | -.073 | .642 |
|  | None | -.059 | .707 |
