# Supplementary material for: Brain–body interactions associated with the transition from mind wandering to awareness of its occurrence
Source: Neurosci Conscious. 2025 Dec 15;2025(1):niaf059. doi: 10.1093/nc/niaf059 (PMC12704443; doi:10.1093/nc/niaf059)
Supplement: Supplementary_Table_3_niaf059 [file supplementary_table_3_niaf059.docx]

**Supplementary Table 3. Statistics on the main effects of temporal aspects on RR intervals**

| **Comparison** | **estimate** | **SE** | **df** | **t-value** | **p-value** | **d** |
| --- | --- | --- | --- | --- | --- | --- |
| Past vs Present | -4.78e-05 | 0.00800 | 254 | -0.006 | 1.0000 | -7.53e-04 |
| Past vs Future | 6.49e-03 | 0.00779 | 254 | 0.833 | 0.8388 | 0.10 |
| Past vs Unknown | -1.93e-02 | 0.00878 | 255 | -2.193 | 0.1279 | -0.27 |
| Present vs Future | 6.54e-03 | 0.00802 | 254 | 0.815 | 0.8473 | 0.10 |
| Present vs Unknown | -1.92e-02 | 0.00895 | 255 | -2.145 | 0.1418 | -0.27 |
| Future vs Unknown | -2.57e-02 | 0.00877 | 255 | -2.937 | .0188 | -0.37 |
