# Supplementary material for: Brain–body interactions associated with the transition from mind wandering to awareness of its occurrence
Source: Neurosci Conscious. 2025 Dec 15;2025(1):niaf059. doi: 10.1093/nc/niaf059 (PMC12704443; doi:10.1093/nc/niaf059)
Supplement: Revised_supplementary_niaf059 [file revised_supplementary_niaf059.docx]

# Supplementary Materials

# Results

## *Mind wandering frequency in each task condition*

We assessed how the frequency of MW reports and the types of thought content varied across task conditions. Although the frequency of thought types during MW has been reported in many studies, the results differ depending on the method used (Banks et al., 2016; Bertossi & Ciaramelli, 2016; McCormick et al., 2018; Spronken et al., 2016; Vannucci et al., 2017). We presented the frequency of thoughts in our experiment to improve our understanding of the nature of MW.

We examined the thought content during MW and identified a significant main effect of emotional valence (*F*(2, 200.85) = 28.0133, *p* < .001, $\eta_{p}^{2}$ = .22), with neutral, positive, and negative thoughts occurring more frequently in that order (Supplementary Table 1 and Supplementary Fig. 1A). Additionally, a main effect of time was observed (*F*(3, 260.71) = 26.3036, *p* < .001, $\eta_{p}^{2}$ = .23), indicating a predominance of thoughts related to the past and future over thoughts related to the present or thoughts without specific temporal features, with past-oriented thoughts being notably frequent (Supplementary Table 1 and Supplementary Fig. 1B).

***Interoceptive accuracy and mind wandering tendencies***

In our study, in addition to exploring brain-body interactions on awareness, we investigated the relationships between MW and interoceptive accuracy (IAcc), an objective accuracy metric for evaluating the detection of internal bodily sensations (Garfinkel et al., 2015). We measured the IAcc via a heartbeat counting task (HCT). A Pearson correlation analysis was conducted to assess the correlation between the frequency of reported thoughts and the IAcc values, adjusting for task conditions (Supplementary Table 2). The findings revealed no significant correlation between the IAcc values and the thought content or MW frequency in this study.

***Mind wandering contents and physiological indices***

The secondary aim of our study was to investigate the influence of thought content on the physiological state and the HEP. We examined the influence of the thoughts' emotional valence (Negative, Neutral, Positive) on RR intervals. Neither the main effect of emotion (*F*(2, 197.28) = 0.577, *p* = .5621, $\eta_{p}^{2}$ = .00582), the main effect of task condition (*F*(1, 197.43) = 2.645, *p* = .105, $\eta_{p}^{2}$ = .01), nor their interaction (*F*(2, 197.36) = 0.2489, *p* = .779, $\eta_{p}^{2}$ = .00252) reached significance. We also examined the effects of thought's temporal aspect (Past, Present, Future, None) and task condition on RR intervals. The results revealed significant main effects of both temporal aspect (*F*(3, 254.62) = 3.005, *p* = .030, $\eta_{p}^{2}$= .03) and task condition (*F*(1, 254.63) = 7.903, *p* = .005, $\eta_{p}^{2}$ = .03). The interaction between these factors was not significant (*F*(3, 254.38) = 2.160, *p* = .093, $\eta_{p}^{2}$= .02).

Subsequent analyses of the main effects, with p-values adjusted for multiple comparisons, revealed two key findings. First, for the main effect of task condition, heartbeats were significantly slower in the BF condition than in the SF condition (*t*(254)=2.811, *p*=.005, *d* = .35). Furthermore, for the main effect of temporal aspect, heartbeats were slower when thoughts had no specific temporal aspect than when they were future-oriented (*t*(255)=−2.937, *p*=.019, d = -0.37). No other pairwise comparisons for temporal aspect were significant (Supplementary Table 3.).

To investigate how the content of thought relates to the central processing of cardiac signals, we performed two separate cluster-based permutation tests on the HEP data, one for the dimension of emotional valence and another for temporal aspects of thought contents. However, neither the analysis of temporal aspects nor that of emotional valence revealed any significant differences.

# Discussion

In our discussion, we proposed that the physiological changes and the perception of such changes led to awareness of MW. While it might be expected that individuals with higher IAcc scores would be more aware of MW and thus report MW more frequently, our results did not support this hypothesis. This inconsistency may occur due to an unconscious transition from MW to an aware state, which is proposed in our framework. According to our model, over-engagement of the MW state occurs before transitioning to the aware state, a shift that does not rely on signals from the body. This mediation of leaving the MW state may be related to the lack of correlation between IAcc scores and the awareness of MW. These differences also highlight that interoception is especially vital to conscious awareness.

Additionally, the lack of a relationship between MW content and IAcc scores, as derived from behavioral metrics, could be due to the nature of these indicators. The frequency of thoughts as an individual measure might reflect not only awareness tendency but also the inherent frequency of MW, which is unique to each individual. Therefore, confounding may occur, as it is challenging to determine whether an individual inherently experiences less MW or lacks awareness of it. Additionally, while many studies have used the HCT to evaluate IAcc scores, a recent study highlighted the limitations of this task (Zamariola et al., 2018). One of the main problems is its narrow focus on attention to heartbeat, which may not reflect general interoception. Refinement of these measures could offer more precise insights into the link between awareness and interoception.

The decoupling hypothesis suggests that engagement in internal, task-unrelated thoughts reduces the processing of external stimuli during task performance (Smallwood & Schooler, 2006). Studies examining this hypothesis on the basis of the brain's oxygen metabolic energy suggest that the brain's overall energy use is limited, with resources used for MW increasing and decreasing when the perceptual load is low and high, respectively (Bruckmaier et al., 2023). Despite advancements in delineating the impact of MW on external stimulus processing, how interoception and thoughts interact remains underexplored. The present study revealed that the processing of cardiac activity was reduced during MW compared to breath focused state (see Main Text, Fig. 3B), indicating that interoceptive signals may serve as unique perceptual modalities and that the processing of this information may vary during MW.

We further performed supplementary analyses to investigate whether this processing during MW was modulated by the content of thought. However, unlike previous research demonstrating that the central processing of cardiac signals differs depending on spontaneous thought contents (Babo-Rebelo et al., 2016; Ito et al., 2019), our analyses did not reveal any significant differences related to thought content. In the present study, thought content relied on participants' spontaneous reports, which led to an imbalanced number of trials across the different content categories. Therefore, it is possible that this relationship could not be fully examined in the current study. Future research using larger datasets or designs that better control for the distribution of thought content is needed to clarify this point.

**Supplementary references**

Babo-Rebelo, M., Wolpert, N., Adam, C., Hasboun, D., & Tallon-Baudry, C. (2016). Is the cardiac monitoring function related to the self in both the default network and right anterior insula? Philosophical Transactions of the Royal Society of London. Series B, Biological Sciences, 371(1708). <https://doi.org/10.1098/rstb.2016.0004>

Banks, J. B., Welhaf, M. S., Hood, A. V. B., Boals, A., & Tartar, J. L. (2016). Examining the role of emotional valence of mind wandering: All mind wandering is not equal. *Consciousness and Cognition*, *43*, 167–176.

Bertossi, E., & Ciaramelli, E. (2016). Ventromedial prefrontal damage reduces mind-wandering and biases its temporal focus. *Social Cognitive and Affective Neuroscience*, *11*(11), 1783–1791.

Bruckmaier, M., Albrecht, V., Tachtsidis, I., & Lavie, N. (2023). On the coupling and decoupling of mind wandering and perception: a shared metabolism account. *Cerebral Cortex Communications*, *4*(4), tgad021.

Garfinkel, S. N., Seth, A. K., Barrett, A. B., Suzuki, K., & Critchley, H. D. (2015). Knowing your own heart: distinguishing interoceptive accuracy from interoceptive awareness. *Biological Psychology*, *104*, 65–74.

Ito, Y., Shibata, M., Tanaka, Y., Terasawa, Y., & Umeda, S. (2019). Affective and temporal orientation of thoughts: Electrophysiological evidence. Brain Research, 1719, 148–156.

McCormick, C., Rosenthal, C. R., Miller, T. D., & Maguire, E. A. (2018). Mind-Wandering in People with Hippocampal Damage. *The Journal of Neuroscience: The Official Journal of the Society for Neuroscience*, *38*(11), 2745–2754.

Smallwood, J., & Schooler, J. W. (2006). The restless mind. *Psychological Bulletin*, *132*(6), 946–958.

Spronken, M., Holland, R. W., Figner, B., & Dijksterhuis, A. (2016). Temporal focus, temporal distance, and mind-wandering valence: Results from an experience sampling and an experimental study. *Consciousness and Cognition*, *41*, 104–118.

Vannucci, M., Pelagatti, C., & Marchetti, I. (2017). Manipulating cues in mind wandering: Verbal cues affect the frequency and the temporal focus of mind wandering. *Consciousness and Cognition*, *53*, 61–69.

Zamariola, G., Maurage, P., Luminet, O., & Corneille, O. (2018). Interoceptive accuracy scores from the heartbeat counting task are problematic: Evidence from simple bivariate correlations. *Biological Psychology*, *137*, 12–17.

**Supplementary Table 1. Statistics on differences in the frequency of thought types**

**Supplementary Table 2. Statistics on the correlation between interoceptive accuracy and the frequency of thought types**

**Supplementary Table 3. Statistics on the main effects of temporal aspects on RR intervals**

**Supplementary Fig. 1. Frequency of MW reports during the task**

**A, B. Thought content frequency in different task conditions:** The vertical axis shows the frequency of MW reports, and the horizontal axis shows the thought content. Each dot represents the data for one participant. **p* < .05, ** *p* < .01, *** *p* < .001.
